# Factors associated with HIV status disclosure to partners and its outcomes among HIV-positive women attending Care and Treatment Clinics at Kilimanjaro region, Tanzania

**DOI:** 10.1371/journal.pone.0211921

**Published:** 2019-03-13

**Authors:** Damian J. Damian, Diana Ngahatilwa, Hatibu Fadhili, Johnston G. Mkiza, Michael J. Mahande, James S. Ngocho, Sia E. Msuya

**Affiliations:** 1 Department of Epidemiology and Biostatistics, Institute of Public Health, Kilimanjaro Christian Medical University College (KCMUCo), Moshi, Tanzania; 2 Community Health Department, Kilimanjaro Christian Medical Centre (KCMC), Moshi, Tanzania; 3 Kilimanjaro Christian Medical University College (KCMUCo), Moshi, Tanzania; 4 Department of Community Health, Institute of Public Health, Kilimanjaro Christian Medical University College (KCMUCo), Moshi, Tanzania; Sefako Makgatho Health Sciences University, SOUTH AFRICA

## Abstract

**Background:**

Sub Saharan Africa continues to be the epicenter of HIV with 70% of people living with HIV globally. Women form nearly 60% of those living with HIV. Studies have shown disclosure of one’s HIV status is important in HIV prevention, in increasing partners who are tested and getting into care early as well as in improving retention in PMTCT and ART programs. This study aimed to determine the prevalence, factors and outcomes of HIV status disclosure to partners among HIV-positive women attending HIV care-and-treatment clinics (CTCs) at Kilimanjaro region, northern Tanzania.

**Methods:**

A cross-sectional study was conducted from January to June 2014 in 3 out of the 7 districts of Kilimanjaro region. The study population was HIV-positive women aged 15–49, who were attending for routine HIV care at 19 selected clinics. Face-to-face interviews were conducted with consenting women to collect necessary information. Multivariate logistic regression analyses were used to determine the independent predictors of HIV status disclosure to partner.

**Results:**

A total of 672 HIV-positive women in Moshi municipal, Hai and Mwanga districts were enrolled. Of them, 609 HIV-positive women reported to have a regular partner. Prevalence of serostatus disclosure to partners was 66%. Of the 400 who had disclosed; 56% did so within the first month of knowing their HIV status. In a multiple logistic regression model, HIV serostatus disclosure was higher among women who: were married/cohabiting (AOR = 4.16, 95% CI: 2.39–7.25; *p*<0.001), currently on ART (AOR = 2.06, 95% CI: 1.11–3.82; *p* = 0.020), and who reported had ever communicated with partners on number of children (AOR = 1.85, 95% CI: 1.15–2.98; *p* = 0.010) and contraceptives use (AOR = 2.01, 95% CI: 1.27–3.20; *p* = 0.208). Most of the women (81%) who disclosed their HIV status to did not reported negative outcomes.

**Conclusion:**

In this setting still a third of the HIV-positive women (34%) fail to disclose their HIV- serostatus to partners. Interventions to impart skills in communication and negotiation between partners may help in improving disclosure of HIV. Efforts to involve men in general sexual and reproductive health including couple counseling and testing will contribute in improving disclosure and communication on HIV among partners.

## Introduction

HIV continues to be a major global public health issue especially in Sub Saharan Africa (SSA). Nearly 70% of the 37 million people living with HIV (PLWHIV) globally at the end of 2016 and 66% of the 1.9 million new infections were in SSA [1]. The global target is to reduce new HIV infections from 1.9 million in 2016 to less than 500,000 by 2020 and to less than 200,000 by 2030 [2]. In order to achieve this, UNAIDS has set the 90-90-90 goal that has to be achieved by 2020. The goal stipulates that, by 2020 we should be able to diagnose 90% of all HIV-positive persons, provide antiretroviral therapy (ART) for 90% of those diagnosed, and achieve viral suppression for 90% of those treated [3]. In Eastern and Southern Africa where Tanzania is located, by the end of 2016, only 62% of PLWHIV knew their status, 54% were on ART and 45% were virally suppressed [2, 3]. Hence efforts for prevention, control and retention into care needs to be strengthened. Tanzania with a total population of 44 million has approximately 1.5 million people living with HIV, with adult HIV-prevalence of 5.1% [4, 5].

Disclosure of one’s HIV status is a key aspect in HIV prevention and treatment programs. It is acknowledged that the process of disclosure of one’s HIV status is complex, not easy, and health providers’ working with PLWHIV therefore needs to give appropriate counseling, support and skills that will enable them to share results with their partners or significant others [6, 7]. Counselors need also to help them to assess the anticipated outcomes and how to deal with them in case they arise. Despite the challenges, several studies have shown advantages of HIV serostatus disclosure to one’s partner [7–12]. Disclosure allow couples to engage in discussion that leads partners of infected individuals to take HIV testing thus enter into care in timely manner [7, 13, 14]. It also gives an opportunity to discuss about condom and contraceptive use that reduce transmission probability and it enable couples to make informed reproductive health choices that may ultimately lower the number of unintended pregnancies [13–15]. Studies have shown that women who disclose to partners have higher ART adherence especially in PMTCT programs, higher adhere to infant feeding method chosen, use contraceptives/condoms more, have lower rates MTCT of HIV, have higher CD4 counts and are retained into care more than those who do not disclose [8, 9, 12, 13, 15]. Understanding, acceptance, moral and emotion support are other reported advantages of disclosure [10, 11].

Prevalence of HIV disclosure to partners vary between settings and populations. Studies from Europe and America reported HIV-serostatus disclosure ranging from 75–85% [10, 16]. In SSA, disclosure ranged from 40%-69% in different countries and populations [9, 15, 17–25]. Despite moderate levels of disclosure in SSA, some women report they fear to disclose due to fear of being rejected and abandoned, fear of being blamed, fear of being considered unfaithful, fear of physical abuse, fear of divorce and stigma [10, 11, 24]. Others reported fear of violence from partners as a barrier [11, 22]. Despite these barriers, studies have shown positive outcomes are predominant compared to negative events [10, 20, 23], hence the need to continue to promote and monitor disclosure in HIV preventive and treatment programs.

At the time of planning this study, most of the studies in Tanzania that had reported on HIV serostatus disclosure, were on pregnant population [9, 18, 20]. Prevalence of disclosure in HIV pregnant women has been shown to be lower than in general population probably due to short period they must make multiple decisions [10]. Longer duration of knowing one’s status and acceptance may influence disclosure [12, 25]. Published information on factors influencing serostatus disclosure among HIV-positive women who are not pregnant and associated outcomes are limited in Tanzania. This study aimed to determine the prevalence, factors and outcomes of HIV serostatus disclosure to partners among HIV positive women aged 15–49 years attending Care and Treatment Clinics in Kilimanjaro region, situated in northern Tanzania.

## Methods

### Study design and sites

The study design was a cross sectional study, conducted from January to June 2014. It was conducted in three out of seven districts of Kilimanjaro region. The districts were Moshi municipality (capital of the region), Hai and Mwanga. Moshi municipality was purposively selected to represent an urban district while the other two were randomly selected out of the remaining six districts to represent rural districts. Kilimanjaro region has a total population of 1,640,087 people and approximately 210,533 live in Hai, 131,442 in Mwanga and 184,292 in Moshi municipal [5].

Regional HIV prevalence is estimated to be 3.8%. That of individual districts are; Hai 5.6%, Moshi municipal 3.6% and Mwanga 4.2% [4].

At the time of data collection Moshi Municipality had a total of 54 health facilities, out of these 9 facilities had HIV ART care and treatment clinics. In Mwanga district, there were a total of 43 facilities out of which 5 had HIV care and treatment services. Hai district had a total of 44 health facilities, 6 had HIV CTC care. All the 20 health facilities (7 hospitals, 9 health centers and 4 dispensaries) with HIV ART care and treatment were eligible to participate in the study. During the study one dispensary was not actively offering ART care and was removed, thus 19 facilities were involved in the study.

### Population and enrolment procedures

The study population was all HIV positive women aged 15–49 who were attending for routine care at the 19 facilities with care treatment clinics (CTCs) in Moshi Urban, Hai and Mwanga districts during the study period. After obtaining an informed consent, face to face interviews were conducted with women at the respective clinics.

### Data collection procedures

Data were collected using a questionnaire developed by the researchers during the face-to-face interview. Pre-testing of data collection tool were done in CTC clinics not selected for this study to ensure reliability and consistency of the tool before the actual data collection started. The interviews were conducted by six trained research assistants (nurses and medical students), who underwent 2 days training. The questionnaire included questions on socio-demographic characteristics (age, education, marital status, partnership status for those who were currently not in union, time in partnership or relationship, employment, income, resident, partners age, education and occupation), questions on reproductive health (number of pregnancies, number of children, desire for more children and contraceptive use), on sexual behavior characteristics (sexual active, number of partners in past 12 months and life time, condom use), questions on clinical characteristics (duration since diagnosis, CD4 count, ART treatment and duration in treatment clinic at diagnosis), and questions on partner communication (ever discussed with partner on number of children, on contraceptive use, on condom use and on HIV testing).

Regarding the outcome variable, women were asked on disclosure of HIV serostatus to partners, time from knowing her status and sharing the results with her partner as well as on outcomes after disclosure. Those who had not disclosed were further asked reasons for failure to disclose and if they have plan to disclose in future. A partner in this study was defined as a person (male) who has been in a steady sexual relationship with a participant for at least 3 months.

### Statistical analysis

Data were entered and analyzed using SPSS statistical software, version 24.0 (SPSS, Chicago, IL, USA). Descriptive statistics were used to summarize the data. Bivariate analysis was used to examine associations between the dependent variable (HIV disclosure to partner) and explanatory variables. Variables found to be significant in the bivariate analyses were entered in the multiple logistic regression analysis to obtain independent predictors of HIV serostatus disclosure to partner.

### Ethical approval and consent to participate

Ethical approval for the study was obtained from KCMU College Ethics Committee prior to commencement of the study. Permission to conduct the study was also sought from District Medical Officers (DMOs) of respective districts. Permission letters from DMOs were presented to all facilities leaders and heads of CTC clinics to inform them on the study and request their permission to collect data. Written informed consent was obtained from every participant, and thumb print was used for those who could not write. Only numbers were used on the questionnaire to maintain confidentiality.

## Results

### Response rate

**Fig 1:**
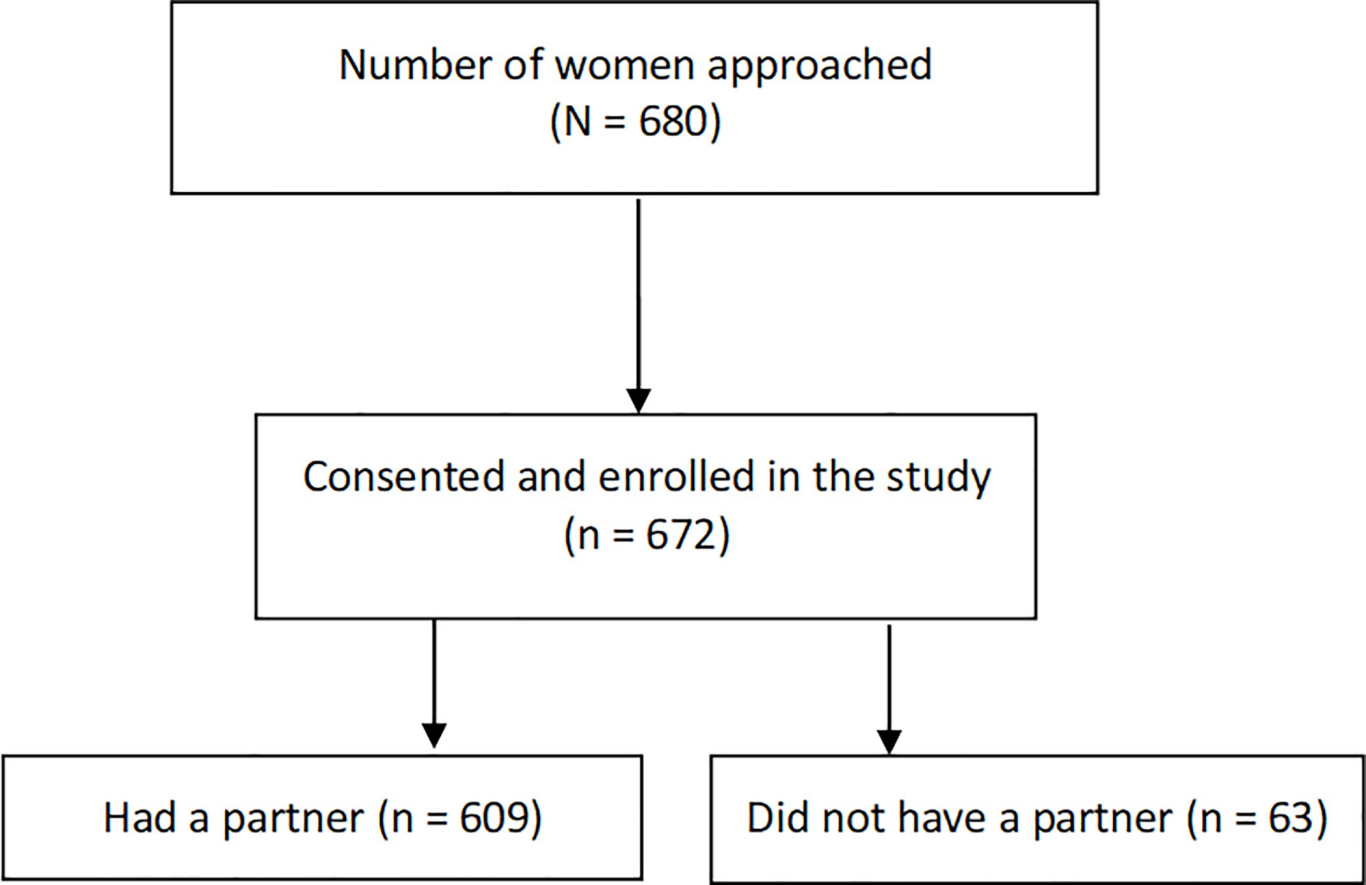
Flow diagram of distribution of the participants.

A total of 680 HIV-positive women aged 15–49 who met inclusion criteria were approached and invited to participate. Of the 680, 672 agreed to participate giving a response rate of 98.8% (Fig 1). However, the analysis is limited to 609 HIV-positive women who reported to have a regular partner. Of the 609; 291 (48%) were enrolled from Moshi municipal, 160 (26%) from Mwanga and 158 (26%) from Hai district respectively. Regarding the level of facility; 336 (55%) of the 609 were enrolled from hospitals, 260 (43%) were enrolled from the health centers and the rest 13 (2%) were enrolled from dispensary.

### Background characteristics of the participants

The mean age of the 609 participants was 36.2 (SD ± 7.6) years. Most of the women had primary education (77%), were not employed (88%), and were married or cohabiting (50%), Table 1.

**Table 1:** Demographic and reproductive health characteristic associated with HIV-status disclosure in Kilimanjaro (N = 609).

Variables	n (%) *	Disclosed HIV status n (%)	OR (95% CI)
**Age**			
< 25 years	48 (7.9)	23 (47.9)	1
≥ 25 years	561 (92.1)	377 (67.2)	2.23 (1.23–4.03)
**Education of the women**			
None	18 (3.0)	12 (66.7)	1
Primary	468 (76.8)	316 (67.5)	1.04 (0.38–2.82)
Secondary or higher	123 (20.2)	72 (58.5)	0.71 (0.25–2.00)
**Marital status**			
Single	102 (16.7)	43 (42.2)	1
Separated/divorced/widowed	202 (33.2)	108 (53.5)	1.58 (0.97–2.55)
Married/ cohabiting	305 (50.1)	249 (81.6)	6.10 (3.74–9.94)
**Employed in past 12 months**			
No	538 (88.3)	355 (66.0)	1
Yes	71 (11.7)	45 (63.4)	0.89 (0.53–1.49)
**Have children**			
No	50 (8.2)	22 (44.0)	1
Yes	559 (91.8)	378 (67.6)	2.66 (1.48–4.78)
**Number of living children (N = 562)**			
≤ 3	414 (68.0)	267 (64.5)	1
> 3	148 (32.0)	113 (76.4)	1.78 (1.16–2.73)
**Sexually active within 12 months**			
No	141 (23.2)	71 (50.4)	1
Yes	468 (76.8)	329 (70.3)	2.33 (1.59–3.43)
**Ever used condoms**			
No	175 (28.7)	96 (54.9)	1
Yes	434 (71.3)	304 (70.0)	1.92 (1.34–2.76)
**Ever use of modern contraceptive**			
No	79 (13.0)	39 (49.4)	1
Yes	530 (87.0)	361 (68.1)	2.19 (1.36–3.53)
**District enrolled**			
Moshi urban	291 (47.8)	181 (62.2)	1
Hai	158 (25.9)	99 (62.7)	1.02 (0.68–1.52)
Mwanga	160 (26.3)	120 (75.0)	1.82 (1.19–2.80)

*Column percent; OR—Odds ratio.

The number of living children the participants had ranged from 0 to 8, with median of 3 children. At the time of the study 42 women (7%) were pregnant. Of the 567 women who were not pregnant, most 416 (73%) reported they do not intend to have more children. However only 235 (66%) of those who did not want children anymore were using modern contraceptives on the day of the interview.

Most of the HIV positive women (66%) were sexually active in past 3 months. A total of 434 (71%) reported to have ever used condoms and out of these 341 (78.6%) reported currently using condoms during sex with their partners. Other background characteristics of the participants are shown in Table 1

#### Partners characteristics and communication

Age of the partners among 428 women who knew the age of their partner ranged from 17–80 years with median of 41 years. Nearly half (48%) had never discussed with the partner about HIV testing, whereas 41% and 52% respectively reported that they have never discussed with their partners on condom use or number of children they should have(Table 2).

**Table 2:** Partners and couple communication characteristics associated with disclosure in Kilimanjaro (N = 609).

Variable	n (%) *	Disclosed HIV status n (%)	Odds ratio (95% CI)
**Age of the partner (N = 428)**			
17–34 years	96 (22.4)	61 (63.5)	1
35–49 years	243 (56.8)	178 (73.3)	1.57 (0.95–2.60)
> 49 years	89 (20.8)	69 (77.5)	1.98 (1.03–3.79)
**Education of the partner (N = 441)**			
Primary	290 (65.8)	214 (73.8)	1
Secondary	121 (27.4)	85 (70.2)	0.84 (0.52–1.34)
Tertiary level	30 (6.8)	17 (56.7)	0.46 (0.22–1.00)
**Ever discussed on number of children with partner (N = 589)**			
No	309 (52.5)	165 (53.4)	1
Yes	280 (47.5)	231 (82.5)	4.11 (2.81–6.02)
**Ever discussed on contraceptive use with partner (N = 585)**			
No	286 (48.9)	148 (51.7)	1
Yes	299 (51.1)	245 (81.9)	4.23 (2.91–6.16)
**Ever discussed on condom use with partner (N = 584)**			
No	238 (40.8)	124 (52.1)	1
Yes	346 (59.2)	269 (77.7)	3.21 (2.24–4.60)
**Ever discussed on HIV testing with partner (N = 585)**			
No	280 (47.9)	129 (46.1)	1
Yes	305 (52.1)	265 (86.9)	7.75 (5.16–11.7)

*Column percent

#### Clinical characteristics of the participants

The median duration of knowing the HIV status was 4 years (IQR: 2–7 years). Of the 609 women, 89% were using antiretroviral medications at the time of interview, with median duration of ART use of 3 years (IQR: 1–6 years). Table 3 depicts other clinical characteristics of the participants.

**Table 3:** Clinical characteristics associated with HIV-status disclosure in Kilimanjaro (N = 609).

Variable	n (%) *	Disclosed HIV status n (%)	Odds ratio (95% CI)
**Time since HIV diagnosis**			
≤ 1 year	138 (22.7)	82 (59.4)	1
2–4 years	191 (31.4)	124 (64.9)	1.26 (0.80–1.99)
5+ years	271 (44.5)	191 (70.5)	1.63 (1.06–2.50)
**Currently on ART treatment**			
No	67 (11.0)	36 (53.7)	1
Yes	542 (89.0)	364 (67.2)	1.76 (1.06–2.94)
**Duration on ART (N = 542)**			
≤ 1 year	156 (25.9)	99 (63.5)	1
2–4 years	186 (30.5)	125 (67.2)	1.18 (0.75–1.85)
5+ years	200 (32.8)	140 (70.0)	1.34 (0.86–2.10)
**Place of initial HIV diagnosis**			
Others	17 (2.8)	8 (47.1)	1
VCT	322 (52.9)	194 (60.2)	1.71 (0.64–4.53)
PMTCT/ ANC	270 (44.3)	198 (73.3)	3.09 (1.15–8.33)
**Ever received FP counseling at CTC**			
No	259 (42.5)	155 (59.8)	1
Yes	350 (57.5)	245 (70.0)	1.57 (1.12–2.19)
**Ever given FP methods at CTC**			
No	312 (51.2)	185 (59.3)	1
Yes	297 (48.8)	215 (72.4)	1.80 (1.28–2.53)

*Column percent

### Prevalence of HIV serostatus disclosure to partner

The proportion of women who had disclosed their HIV serostatus to their partners was 65.7% (400/609). Time to disclosure to partners ranged from 0–206 months. More than half (56%) of those who disclosed did so within the same month of receiving the HIV results, (27%) disclosed between 2^nd^ and 12^th^ month; 10% disclosed between 13–60 months after receiving the results and 7% disclosed after 60^th^ month [> 5 years] of knowing the test results.

### Factors associated with HIV serostatus disclosure to partner

#### Bivariate analysis

District, age, marital status, sexual activity and condom use were significantly associated with HIV-serostatus disclosure in the bivariate analysis. Women enrolled from Mwanga district had 82% higher odds of HIV-serostatus disclosure to partners than those in Moshi urban district (OR = 1.82, 95% CI: 1.19–2.80). Women who were 25 years or older had 2-fold increase in odds of HIV-serostatus disclosure to partners than those who were younger than 25 years (OR = 2.23, 95% CI: 1.23–4.03). Those who were married/ cohabiting had 6-fold increase in odds of HIV-serostatus disclosure to partners than those who were single (OR = 6.10, 95% CI: 3.74–9.94). Participants who were sexually active within the past 12 months had 2 times high odds of HIV-serostatus disclosure to partners than those who were not sexually active (OR = 2.33, 95% CI: 1.59–3.43). Those who had ever used condoms or contraceptives had significantly 1.92 and 2.19 higher odds of HIV-serostatus disclosure to partners than others respectively i.e. (OR = 1.92, 95% CI: 1.34–2.76) and (OR = 2.19, 95% CI: 1.36–3.53). The associations of socio-demographic and reproductive health characteristics of the participants with disclosure is presented in Table 1.

Table 2 depicts partners characteristics associated with disclosure of HIV status to partners. Women who reported that they have ever discussed with their partners about numbers of children they wanted (OR = 4.11, 95% CI: 2.81–6.02), contraceptive use (OR = 4.23, 95% CI: 2.91–6.16); condom use (OR = 3.21, 95% CI: 2.24–4.60) and HIV testing (OR = 7.75, 95% CI: 5.16–11.70) had significantly higher odds of disclosure compared to those who never discussed with their partners. Table 3 presents clinical characteristics associated with HIV sero-status disclosure to partners. Women who were initially diagnosed at PMTCT/ANC had 3 times higher odds of disclosure than those who were diagnosed at VCT or elsewhere (OR = 3.09, 95% CI: 1.15–8.33). Women who knew their HIV diagnosis for 5+ years had 63% higher odds of disclosure than those who knew for ≤ 4 years (OR = 1.63, 95% CI: 1.06–2.50). Furthermore, women who were on ART treatment had 76% significantly higher odds of HIV-serostatus disclosure to partners than those who were not on treatment (OR = 1.76, 95% CI: 1.06–2.94). Those who received FP counselling at CTC and those given FP methods at CTC had more than 50% higher of odds of HIV-serostatus disclosure to partners than those who did not i.e (OR = 1.57, 95% CI: 1.12–2.19) and (OR = 1.80, 95% CI: 1.28–2.53) respectively.

#### Multivariate analysis

Factors independently associated with HIV sero-status disclosure to partners are presented in Table 4. Marital status, current ART status, and ever discussed with partners on number of children or contraceptive use remained significantly associated with HIV-serostatus disclosure to partner. Women who were married/cohabiting had 4-fold increase in odds of HIV-serostatus disclosure to partners than those who were single (AOR = 4.16, 95% CI: 2.39–7.25; *p*<0.001)Those on ART treatment had 2 times higher odds of HIV-serostatus disclosure to partners than those who were not on treatment (AOR = 2.06, 95% CI: 1.11–3.82; *p* = 0.020). Women who reported discussing with their partners about numbers of children they wanted had 85% higher odds of HIV-serostatus disclosure to partners than those who did not (AOR = 1.85, 95% CI: 1.15–2.98; *p* = 0.010). Those who reported discussing with their partners on contraceptive use had 2-fold increase in odds of HIV-serostatus disclosure to partners than those who did not (AOR = 2.01, 95% CI: 1.27–3.20; *p* = 0.208).

**Table 4:** Factors independently associated with HIV status disclosure among partners in Kilimanjaro.

Variable	AOR (95% CI)
**Age**	
< 25 years	1
≥ 25 years	1.61 (0.78–3.34)
**Marital status**	
Single	1
Separated/divorced/widowed	1.38 (0.80–2.37)
Married/ cohabiting	4.16 (2.39–7.25)
**On ART**	
No	1
Yes	2.06 (1.11–3.82)
**Ever used condoms**	
No	1
Yes	1.54 (0.99–2.38)
**Discussed on number of children**	
No	1
Yes	1.85 (1.15–2.98)
**Discussed on contraceptive use**	
No	1
Yes	2.01 (1.27–3.20)
**Level of facility enrolled**	
Hospital	1
Health Centre	1.29 (0.86–1.94)
**Place of initial HIV diagnosis**	
Others	1
VCT	1.04 (0.32–3.37)
PMTCT/ ANC	1.61 (0.49–5.28)

AOR- Adjusted Odds ratio

### Outcomes of HIV serostatus disclosure to partner

Of the 400 HIV positive women who disclosed, the majority 81% (325) reported positive outcomes i.e. women reported that their partners were fine and understanding; either they accepted the situation or did not do anything. About 4.5% reported their partners wanted to go and get tested immediately and it helped discussions on using of condoms. Some reported negative events. A total of 43 (10.5%) reported that disclosure resulted into fights or blame from their partners; 7.3% reported that their partners either run away or left them and 3.3% reported their partners were confused and depressed.About one in four women (24.7%) of those who did not disclose their HIV serostatus to their partners were afraid that once they disclosed their partners might run away and leave them or divorce them. Few (3.3%) did not disclose because they were afraid of the fight or that their partners may harm them.

## Discussion

Prevalence of disclosure to partners among HIV-positive women aged 15–49 years attending CTC in 3 districts of Kilimanjaro region was 66%. The prevalence of HIV sero-status disclosure to partner observed in this study was higher compared to that of pregnant women in Dar es Salaam (44%) and HIV positive women in Morogoro, Tanzania (41%) [9, 18]. Moreover, it was higher than that observed in South Africa among HIV positive pregnant women (59%), in Makonde, Zimbabwe ^(^55%),in Abidjan, Ivory coast (46%) and in Burkina Faso (17.6%) [10, 13, 18, 20]. The disclosure of HIV sero-status to partner among HIV-positive women in Tanzania has observed to increase over time e.g. from 16.7% in 2001 to 66% in 2014 found in the current study [9, 18, 20].

Markers of partner’s communication improved HIV-serostatus disclosure. Findings from this study showed that discussion with partners on number of children, condom use, HIV testing, and on contraceptive use, increased the odds of disclosure of HIV serostatus to partners. Also, women who had ever used condoms or contraceptives had higher odds of disclosure. In Ethiopia Alemayehu et al (2014) and Genet et al (2015) respectively reported that disclosure was higher among women who got pretesting counseling and those who had discussion prior to HIV testing [17, 25]. In Morogoro, Tanzania it was reported that disclosure was higher among pregnant women who ever used condoms and who knew HIV status of their partners [9]. Programs that can empower women by improving their communication skills especially in the issues of sexual and reproductive health, and in use of condoms or contraceptives may be one of the key strategies in improving disclosure. Further strengthening of couple counseling is needed in order to improve proportion of couples who know their status and start ART care early.

Women in stable partnership i.e. married/ cohabiting had higher odds of disclosure than others. This is similar to previous studies conducted in Tanzania, Kenya and Burkina Faso [8, 9, 12–14]. This makes senses because it may be possible that those married or staying together have contact and hence, they are able to disclose as compared to those that are no longer in contact with each other. For providers, this means there is a need to develop other strategies of trying to reach previous partners of those who are not currently in union. These should be developed with inputs from clients themselves.

Most of the women from this study didn’t report negative reaction after disclosure, with 81% of the participants reported positive outcomes after disclosure of HIV sero-status to partner. The positive outcomes were acceptance and understanding of the situation. Some women reported negative reactions such as blame, depression, accusation, divorce and separation from partners. Similar findings were reported in study by other studies in Tanzania where 91.7% they reported positive outcomes after disclosure and 14.6% reported negative outcomes [19]. Maman et al (2003) and Medley et al (2004) also reported that higher rates of positive outcomes should motivate programs to continue to encourage and support HIV-positive people to disclose to partners in order to reduce horizontal HIV transmission and help partners to enter in timely manner in ART care and management.

It is still worrying that 20% of married/cohabiting, and 46% of separated/ divorced have not disclosed their status to partners. Fear of negative events like partners abandoning them or divorce has been reported by 10% of the women who have not disclosed. It is hoped that disclosure of HIV status to partners will prompt partner testing and knowing their status early. This will contribute in reaching the first 90 in the UNAIDS goal of 90-90-90 by 2020 [1, 2]. The regional and district health teams working in HIV prevention and care would need to improvise other means in addition to current methods of informing partners about HIV testing without exposing women to harm.

The limitations of the study were that the study design was cross-sectional, so the temporal nature of the associations cannot be determined. Secondly, the key outcome measure i.e. “*disclosure of HIV-serostatus”* depended on reported information from the women, some women might give information according to what they think the researcher wants and might lead to over or under-reporting on disclosure. Despite these limitations, this study provided information from 3 districts fairly representing HIV positive women in the region.

## Conclusion

Most of the HIV positive women (66%) in 3 districts in Kilimanjaro region disclosed their HIV serostatus to their partners. Communication between partners, ever used condoms and awareness of HIV status of the partner were the factors influencing HIV disclosure to partners.

The study also found that most of the women who disclosure their HIV sero status to their partners didn’t not reported negative outcomes (81%).

However, there is a need to come with different strategies on reaching current and previous partners of people who test HIV-positive and are in care in order to help the country to achieve the goal of 90% of people know their HIV status, 90% of those knowing their status getting into care and become viral suppressed.

## Supporting information

S1 File Questionnaire. (PDF)

S2 File Dataset. (XLSX)

## References

[1] UNAIDS *Global AIDS Epidemic Update 2016* United Nations Programme on HIV/AIDS, Geneva, Switzerland: (Available from: http://www.unaids.org/en/).

[2] UNAIDS *Prevention Gap Report summary*. United Nations Programme on HIV/AIDS, Geneva, Switzerland, 2016.

[3] UNAIDS *AIDS by the numbers. AIDS is not over, but it can be*. UNAIDS; 2016.

[4] National Bureau of Statistics [Tanzania] and ORC Marco. *Tanzania HIV/AIDS and Malaria Indictor Survey (THMIS) 2011/12* Dar es Salaam, Tanzania: NBS and ORC Macro, 2012 Available at: http://www.nbs.go.tz/THIS/THIS2011-12/THIS2011-12.htm.

[5] United Republic of Tanzania. *2012 Population and household census*: *Population distribution by administrative areas* National Bureau of Statistics, Dar es Salaam and Office of Chief Government Statistician, Zanzibar, 3 2013

[6] UNAIDS & WHO: Opening up the HIV/AIDS epidemic: *Guidance on encouraging benefitial disclosure*, *ethical partner counseling and appropriate use of HIV case-reporting* UNAIDS & WHO, 11 2000, Geneva, Switzerland.

[7] Walcott M, Hatcher A, Kwena Z, Twan JM: Facilitating HIV status disclosure for pregnant women and partners in rural Kenya: a qualitative study. *BMC Public Health* 2013, 13: 1115 10.1186/1471-2458-13-1115 24294994 PMC3907031

[8] Kiarie JN, Kreiss JK, Richardson BA, John-Stewart GC: Compliance with antiretroviral regimens to prevent perinatal HIV-1 transmission in Kenya. *AIDS* 2003, 17:65–71. 10.1097/01.aids.0000042938.55529.e1 12478070 PMC3387271

[9] Kiula ES, Damian DJ, Msuya: Predictors of HIV serostatus disclosure to partners among HIV positive pregnant women in Morogoro, Tanzania. *BMC Public Health* 2013, 13: 433 10.1186/1471-2458-13-433 23641927 PMC3668140

[10] Maman S, Mbwambo JK, Hogan NM, Weiss E, Kilonzo GP, Sweat MD: High rates and positive outcomes of HIV-serostatus disclosure to sexual partners: reasons for cautious optimism from a voluntary counselling and testing clinic in Dar es Salaam, Tanzania. *AIDS Behav* 2003, 7: 373–82. 10.1023/b:aibe.0000004729.89102.d4 14707534

[11] Medley A, Moreno C, McGill S, Maman S: Rate, Barriers and Outcome of HIV serostatus disclosure among women in developing countries: Implication for prevention of mother to child transmission programmes. *Bulletin of the World Health Organization* 2004, 80(4). PMC2585956 15259260

[12] Trinn TT, Yatich N, Ngoma R, McGrath CJ, Richardson BA, Sakr SR, Langat A, John-Stewart GC, Chung MH. Partners disclosure and early CD4 response among HIV-infected adults initiating antiretroviral treatment in Nairobi Kenya. *PLoS One* 2016, 11(10): e0163594 10.1371/journal.pone.0163594 27711164 PMC5053490

[13] Msuya SE, Mbizvo EM, Hussain A, Uriyo J, Sam NE, Stray-Pedersen B: Low male partner participation in antenatal HIV counseling and testing in northern Tanzania: implications for preventive programs. *AIDS Care* 2008, 20(6): 700–9. 10.1080/09540120701687059 18576172

[14] Nebie Y, Meda N, Leroy V, Mendelbrot L, Yaro S, Sombie I, Cartoux M, Tiendrebeogo S, Dao B, Ouangre A, Nacro B, Fao P, Ky-Zerbo O, Van de Perre P, Dabis F. Sexual and Reproductive life of women informed of their HIV seropositivity:A prospective cohort study in Burkina Faso. *JAIDS* 2001, 28: 367–372. 11707674 10.1097/00126334-200112010-00010

[15] Elopre L, Hook EW, Westfall AO, Zinski A, Mugavero MJ, Turan J, Van Wagoner N. The role of early HIV status disclosure in retention in HIV care. AIDS Patient Care STDS 2015, 29(1): 646–650. 26588053 10.1089/apc.2015.0205 PMC4684646

[16] Sullivan K, Voss J, Li D: Female disclosure to HIV positive serostatus to sex partners in Hawaii and Washington, USA. *Women Health* 2010, 50(6): 506–526. 10.1080/03630242.2010.516697 20981634 PMC2975569

[17] Alemayehu M, Aregay A, Kalayu A, Yebyo H: HIV disclosure to sexual partner and associated factors among women attending ART clinic at Makelle hospital, Northern Ethiopia. *BMC Public Health* 2014, 14: 746 10.1186/1471-2458-14-746 25056689 PMC4124165

[18] Antelman G, Fawzi MCS, Kaaya S, Mbwambo J, Msamanga GI, Hunter DJ, Fawzi WW: Predictors of HIV-1 serostatus disclosure: a prospective study among HIV-infected pregnant women in Dar-es-salaam, Tanzania. *AIDS* 2001, 15: 1865–1874. 10.1097/00002030-200109280-00017 PMC6261328 11579250

[19] Brou H, Djohan G, Becquet R, Allou G, Ekouevi D, Viho I, Leroy V, Desgrees-du-Lou A, ANRS 1201/1202/1253 Ditrame Plus Study Group: When do HIV-infected women disclose their HIV status to their male partner and why? Studies in a PMTCT programme Abidjan. *PLoS Medicine* 2007, 4(12): 1912–1920. 10.1371/journal.pmed.0040342 PMC2100145 18052603

[20] Kilewo C, Massawe A, Lyamuya E, Semali I, Kalokola F, Urassa E: HIV counseling and testing of pregnant women in sub-Saharan Africa. *AIDS* 2001, 28: 458–462 10.1097/00042560-200112150-00009 11744835

[21] Makin JD, Forsyth BW, Visser MJ, Sikkema KJ, Neufeld S, Jeffery B: Factors affecting disclosure in South Africa HIV positive pregnant women. *AIDS Patient Care and STDs* 2008, 22(11). 10.1089/apc.2007.0194 PMC2929151 19025485

[22] Mcheto P, Chadambuka A, Shamira G, Tshimanga M, Notion G, Nyamayaro W: Determinants of non-disclosure of HIV status among women attending the PMTCT programme in Makonde district, Zimbabwe. *Pan African Medical Journal* 2011, 8: 51 10.4314/pamj.v8i1.71169 PMC3201613 22121458

[23] Norman A, Chopra M, Kadiyala S: Factors related to HIV disclosure in two South African communities. *American Journal of Public Health* 2007, 97(10). 10.2105/AJPH.2005.082511 PMC1994182 17761582

[24] Vu L, Andrinopoulus K, Mathew C, Chopra M, Kendall C, Eisele TP: Disclosure of HIV status to sex partners among infected men and women in Cape Town, South Africa. *AIDS Behav* 2012, 16: 132–138 10.1007/s10461-010-9873-y 21197600

[25] Genet M, Sebsibie G, Guitie T. Disclosure of HIV seropositive status to sexual partners and its associated factors among patients attending antiretroviral treatment clinic follow up at Mekelle Hospital, Ethiopia: a cross sectional study. *BMC Res Notes* 2015, 8: 109 10.1186/s13104-015-1056-5 25889779 PMC4379749

