# Supplementary material for: Factors associated with HIV status disclosure to partners and its outcomes among HIV-positive women attending Care and Treatment Clinics at Kilimanjaro region, Tanzania
Source: PLoS One. 2019 Mar 13;14(3):e0211921. doi: 10.1371/journal.pone.0211921 (PMC6415788; doi:10.1371/journal.pone.0211921)
Supplement: S1 File — (PDF) [file pone.0211921.s001.pdf]

FACTORS ASSOCIATED WITH HIV STATUS DISCLOSURE TO PARTNERS AND ITS OUTCOMES AMONG HIV- POSITIVE WOMEN ATTENDING CARE AND TREATMENT CLINICS AT KILIMANJARO REGION, TANZANIA.

QUESTIONNAIRE

Questionnaire number …………………….. **Date of Interview** ……………………….

Name if interviewer ………………………………………………………………….

Respondent file number ………………………………………………………………...

Name of the health facility ……………………………………………………………

Level of facility: 1. Dispensary, 2. Health Centre, 3. Hospital …………………………

Ownership status: 1. Government, 2. FBO, 3. Private, 4. Parastatal …………………...

District ……………………………………………………………………………....

	QUESTION	ATRIBUTES	CODES
**SECTION A: SOCIO DERMOGRAPHIC INFORMATION**			
1	When were you born?	Date/Month/ year_______________	
2	What is your education level?	Never attended to school<br>Primary education(class)<br>Secondary education<br>Tertiary education	01<br>02<br>03<br>04
3	Within the past 12 months have you been employed in a sector where you received regular monthly salary?<br>If yes, type of job	Yes<br>No<br>…………………………………..	01<br>02
4	If you are not employed, what do you do to earn income?	Big farmer<br>Peasant<br>Business<br>Housewife<br>Others, specify……………….	01<br>02<br>03<br>O4
5	What is you are approximate monthly income?	Mention…………………...TZS	
6	What is your current marital status?	Married, for how long………...<br>Cohabiting, for how long……….<br>Separated, for how long…………	01 go Q7&9<br>02 go Q7&9<br>03 go Q8

		Divorced, for how long…………<br>Widow, for how long…………<br>Single	04 go Q8<br>05 go Q8<br>06 go Q8
7	Are you currently living together or visits?	Live together<br>Visits<br>3-6 months apart<br>7>more months apart	01<br>02<br>03
8	Though you said you are widow, divorced, separated, or single; do you have a regular partner?	Yes<br>No	01 go to Q9<br>02go to Q15
9	How old is your partner?	…………………years	
10	What is his level of education?	Never attended to school<br>Primary education(class)<br>Secondary education<br>Tertiary education	01<br>02<br>03<br>04
11	What is your partner's occupation?	………………………………	
12	Does he have other wives (polygamy)?	Yes<br>No<br>Don't know	01<br>02<br>03
13	Does he have other partners outside your relationship?	Yes<br>No<br>Don't know	01<br>02<br>03

SECTION B: QUESTIONS ON INDIVIDUAL HEALTH			
14	When were you diagnosed with HIV?	………………………. month/year	
15	Where were you diagnosed?	PMTCT/ANC<br>VCT<br>Another place…………..	01<br>02<br>03
16	Do you know your current CD4 count (taken within past 6 months)	Yes<br>No	01<br>02skip Q17
17	If the answer is yes above what is the value	……………………………………….	
18	Are you currently on ARVs	Yes<br>No	01<br>02skip Q19 and Q19b
19	If yes which month and year have you started using ARVs	………………..month/year	
19b	If yes, mention the drugs		
20	Are you taking any other drugs apart from ARV?	Yes<br>No	01<br>02
21	If yes, mention		
**SECTION C: REPRODUCTIVE HEALTH HISTORY**			
22	Do you have children	Yes<br>No	
23	How many times have you ever been pregnant?	………………………………….	
24	Number of living children	……………………………………..	
25	Number of live births	………………………………………	
26	Number of children born alive, but later died	……………………………………	
27	Do all your children have the same father	Yes<br>No	01<br>02
28	Are you currently pregnant?	Yes<br>No	01<br>02
29a	If not pregnant, do you intend to become pregnant/to have more children?	Yes<br>No	01<br>02
29b	If yes, after how long	………………………. year	
30	If does not plan to have more children, why not?		
**SECTION D: NUMBER OF SEXUAL PARTNERS**			

31a	Are you sexually active? i.e. Have you practiced sex within the past 3 months?	Yes<br>No….	01go to Q32<br>02
31b	If no, have you had sex in within the past one year?	Yes<br>No….	01<br>02
31c	Why have you stopped having sex?		
32	Have you ever used condoms?	Yes<br>No………	01<br>02go to Q36
33a	If yes, do you use condom with your current partner?	Yes<br>No	01<br>02
33b	If yes, do you use when you meet/have sex every time.	Yes<br>No	01<br>02
34a	Including your current partner, how many numbers of sexual partners have you had in the past 12 months	Number: ……………………	
34b	Including your current partner, when you include your current partner, in total how many partners have you had during your life time	Number: …………………….	
35	When you lastly had sex with partner, did you use condom?	Yes<br>No	01<br>02
**SECTION E: PARTNER COMMUNICATION AND DISCLOSURE**			
36	Is your partner aware of your HIV status/Does your partner know your HIV status?	Yes<br>No	01<br>02
37	If yes, when did you inform him?	………………………. month/year	
38	What was the reaction of your partner? What did the partner do after disclosing?		
39	If has not disclosed, what does she thinks will be the reaction of the partner if she does tell him?		
40	Do you know the HIV status of your partner?	Yes<br>No	01<br>02
41	If yes what is his status	Positive<br>Negative	01<br>02
42	Have you ever been coupled counseled and tested?	Yes<br>No	01<br>02
